# Exosome Cargo in Neurodegenerative Diseases: Leveraging Their Intercellular Communication Capabilities for Biomarker Discovery and Therapeutic Delivery

**DOI:** 10.3390/brainsci14111049

**Published:** 2024-10-23

**Authors:** Shuai Zhang, Yu Yang, Xinchen Lv, Xue Zhou, Wangqian Zhao, Linfeng Meng, Shaohua Zhu, Zhixiang Zhang, Ying Wang

**Affiliations:** Department of Forensic Medicine, School of Basic Medical Sciences, Soochow University, Suzhou 215123, China; 20224021009@stu.suda.edu.cn (S.Z.);

**Keywords:** neurodegenerative diseases, exosomes, pathophysiology, biomarkers, drug delivery

## Abstract

The inexorable progression of neurodegenerative diseases (NDs), including Alzheimer’s disease, Parkinson’s disease, Huntington’s disease, amyotrophic lateral sclerosis, and multiple sclerosis, is closely related to irreversible brain decline. Accurately characterizing pathophysiological features and identifying reliable biomarkers for early diagnosis and optimized treatment are critical. Hindered by the blood–brain barrier (BBB), obtaining sensitive monitoring indicators for disease progression and achieving efficient drug delivery remain significant challenges. Exosomes, endogenous nanoscale vesicles that carry key bioactive substances, reflect the intracellular environment and play an important role in cell signaling. They have shown promise in traversing the BBB, serving dual roles as potential biomarkers for NDs and vehicles for targeted drug delivery. However, the specific mechanisms by which exosome influence NDs are not fully understood, necessitating further investigation into their attributes and functionalities in the context of NDs. This review explores how exosomes mediate multifaceted interactions, particularly in exacerbating pathogenic processes such as oxidative stress, neuronal dysfunction, and apoptosis integral to NDs. It provides a comprehensive analysis of the profound impact of exosomes under stress and disease states, assessing their prospective utility as biomarkers and drug delivery vectors, offering new perspectives for tackling these challenging diseases.

## 1. Introduction

The normal function of the central nervous system (CNS) fundamentally relies on the exchange and integration of information across complex networks of neurons and glial cells, including microglia, astrocytes, and oligodendrocytes [1]. This cellular communication is essential not only for brain development and homeostasis, but also closely associated with the occurrence of neurodegenerative diseases (NDs), such as Alzheimer’s disease (AD), Parkinson’s disease (PD), Huntington’s disease (HD), amyotrophic lateral sclerosis (ALS), and multiple sclerosis (MS) [1,2]. These diseases are attributed to the progressive deterioration of neuronal structure and functions [3], accompanied by the accumulation of misfolded proteins [4,5], such as amyloid-beta (Aβ) and tau in AD, alpha-synuclein (α-syn) in PD, mutant huntingtin (HTT) in HD, SOD1 and TDP-43 in ALS, and Epstein-Barr virus protein in MS. Common pathological features include oxidative stress [6], neuroinflammation [7], neuronal degeneration [7], and apoptosis [8]. Despite differences in clinical and pathological features, NDs uniformly impair brain function. Early detection is challenging due to the gradual onset of symptoms and the reliance on costly neuroimaging and biochemical examinations of cerebrospinal fluid (CSF). In addition, there is a lack of real-time histological monitoring of brain tissue and effective peripheral sensitive indicators [9]. Consequently, there is an urgent need for novel, specific biomarkers for early detection during the preclinical stage.

The blood–brain barrier (BBB) functions as a critical diffusion barrier that is essential for the normal functioning of the CNS [10]. Research indicates that the majority of molecules, including peptides and genetic drugs, cannot penetrate the BBB. In NDs, although BBB integrity is compromised and its permeability altered, therapeutic drugs still fail to effectively enter the CNS and are restricted to peripheral circulation, thereby increasing the risk of side effects [9,11]. Therefore, the safe and effective transportation of therapeutic drugs across the BBB remains a pivotal challenge in NDs treatment. Current strategies aimed at overcoming BBB selectivity include neurosurgical procedures, biochemical disruption of the BBB, and different nanoparticle formulations [12,13,14]. However, these approaches often face the additional obstacle of rapid clearance by the immune system, limiting their effectiveness and duration of action. In contrast, several studies have demonstrated that exosomes are capable of crossing the BBB to deliver functional cargoes with minimal immune system interaction [15]. Specific proteins on the surface of exosomes can be recognized by integrins or other receptors on target cells, facilitating their evasion of immune detection and promoting transport and cellular fusion [16]. Notably, exosomes can be administered through multiple routes, including nasal, venous, abdominal, and intracranial pathways, thereby enhancing their suitability for use in NDs’ treatment [17,18,19].

Exosomes and their mechanisms of production and function in multicellular organisms cover many fields, from physiological tissue regulation to pathogenic injury and organ remodeling. Recent advancements in this field have been propelled by studies highlighting the association between circulating exosomes and the risk and severity of various diseases, including digestive system diseases [20], cardiovascular diseases [21], cancer [22], and NDs [23]. The role of exosomal cargoes in disease pathology and the potential for modifying their contents position them as promising candidates for biomarkers [24]. In addition, exosomes have received attention as potential therapeutic targets [25], drug delivery vehicles [26], and tools for biomedical applications [27]. Given their peripheral availability, ability to cross the BBB, and targeted delivery capabilities, exosomes are emerging as valuable biomarkers and drug carriers for NDs. Therefore, the delivery efficiency of exosomes and their functional diversity provide new methods for the early detection, diagnosis, and treatment of NDs.

The intersection between exosome biogenesis and the regulation of secretory vesicles in neuronal cells offers new insights into the putative connection between exosomes and the pathogenesis of NDs, biomarker discovery, and therapeutic development and application. However, comprehensive studies on the morphology and function of exosomes derived from patients with NDs remain limited, and their exact role in the pathophysiology of these conditions has not yet been fully elucidated. To evaluate the potential of exosomes for the pathogenesis, diagnosis, and therapy of NDs, we conducted a systematic searched of relevant databases of literature up to June 2024, including PubMed, Embase, and Web of Sciences. The search employed a combination of terms “neurodegenerative diseases” and “exosomes”. A total of 196 relevant articles were identified and included in this review, of which 9 were meta-analyses. This review focuses on exosomes and their physiopathological significance in NDs, providing a detailed examination of exosome biogenesis, secretion, uptake, and function. It also offers a comprehensive overview of exosomes in age-related NDs such as AD, PD, HD, ALS, and MS, highlighting the latest research advancements. Lastly, this review discusses the prospects of exosomes as biomarkers, diagnostic tools, and therapeutic carriers for NDs.

## 2. Exosomes: Biogenesis, Secretion, Uptake, and Function

### 2.1. Biogenesis and Secretion of Exosomes

**Figure 1:**
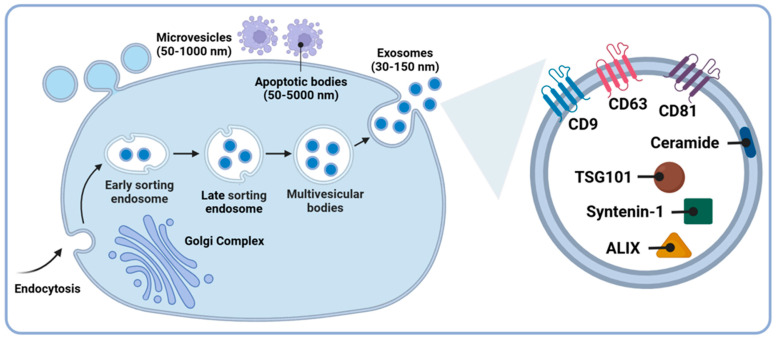
The three major categories of EVs: microvesicles, apoptotic bodies, and exosomes. Microvesicles are released through plasma membrane budding and range in size from 50 nm to 1000 nm. Apoptotic bodies, formed from cells undergoing apoptosis, are expelled as blebs into the extracellular space, with diameters ranging from 50 to 5000 nm. In contrast, exosomes originate in the endosomal pathway, beginning with the formation of early-sorting endosomes, progressing to late-sorting endosomes, and eventually forming multivesicular bodies. Their release into the extracellular space occurs upon the fusion of multivesicular bodies with the plasma membrane, and they range in size from 30 to 150 nm. Additionally, exosomes are identified by specific protein markers, including tetraspanins (CD9, CD81, CD63), syntenin-1, TSG101, ceramides, and ALIX. This figure was created with BioRender.com.

Extracellular vesicles (EVs) are increasingly recognized as significant intercellular communicators. Originally thought to be solely membrane-bound vesicles released through cell membrane budding, EVs are now known to exist in various forms within extracellular spaces, including tissues and biological fluids [28]. Beyond being products of plasma membrane shedding, EVs are also secreted during exocytosis via the fusion of multivesicular bodies (MVBs) with the plasma membrane [29]. This varied intracellular genesis endows EVs with different types, compositions, and functionalities [30]. The primary classes of EVs include apoptotic bodies, microvesicles (MVs), and exosomes (Figure 1), distinguishable by size, biogenesis, and content. Exosomes, the smallest class released by cells, range from 30 to 150 nm in diameter, whereas MVs and apoptotic bodies range from 100 to 1000 nm and from 50 to 5000 nm in diameter, respectively. Unlike apoptotic bodies, which originate from vesicles of dying cells, and MVs, which bud outward directly from the plasma membrane, exosome formation is a highly orchestrated process. It begins with the invagination of the plasma membrane that incorporates cell surface and extracellular components, forming early-sorting endosomes (ESEs). ESEs mature into late-sorting endosomes (LSEs), whose membrane invaginate to form MVBs, ultimately producing intraluminal vesicles (ILVs) that become exosomes. Exosomes are specifically enriched with proteins such as tetraspanins (CD63, CD9, CD81), tumor susceptibility gene 101 (TSG101), syntenin-1, apoptosis-linked gene 2-interacting protein X (ALIX), and ceramides, differentiating them from MVs, apoptotic bodies, and other cellular contaminants [31,32].

**Figure 2:**
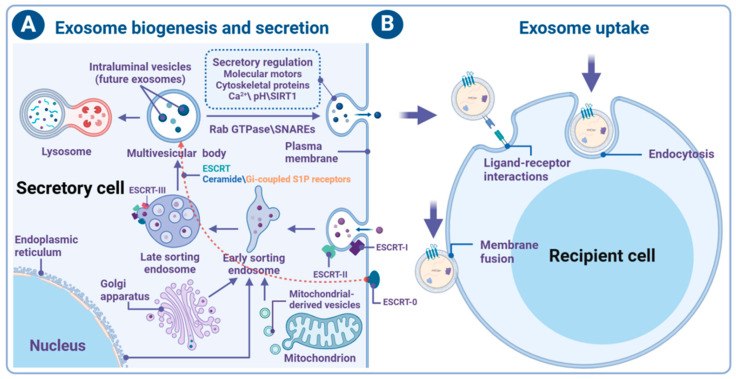
Biogenesis, secretion, and uptake of exosomes. (**A**) Liquids and extracellular components, including proteins, lipids, metabolites, small molecules, and ions, enter the cell by endocytosis and plasma membrane invagination along with cell surface proteins. This budding process leads to the formation of early-sorting endosomes or the possible fusion of the bud with preformed early-sorting endosomes derived from the endoplasmic reticulum, Golgi apparatus, and mitochondria-derived molecular substances or vesicles. A subsequent invagination of late-sorting endosomes generates a set of intraluminal vesicles (future exosomes) of varying sizes and contents that eventually form multivesicular bodies. The assembly of multivesicular bodies is dependent on the ESCRT pathway, characterized by the sequential recruitment of ESCRT machinery proteins and ESCRT-associated proteins to the endosomal membrane. Conversely, multivesicular bodies’ maturation also depends on ceramides and G protein (Gi)-coupled sphingosine 1-phosphate (S1P) receptors in a controlled manner, represented by the dotted red arrow. MVBs may either fuse with lysosomes for degradation or, via an alternate route involving Rab GTPase and SNAREs, proceed to the plasma membrane to release exosomes. The release of exosomes is influenced by various factors, including pH, Ca^2+^, SIRT1, and proteins associated with molecular motors and cytoskeletal. (**B**) The exosomes released into the extracellular space are captured by recipient cells through endocytosis, membrane fusion, ligand-receptor binding, and other pathways, thereby affecting the state of the recipient cells. This figure was created with BioRender.com.

The heterogeneity of exosome cargo depends on highly selective cargo separation and sorting during their formation and is mainly governed by either ESCRT-dependent or -independent pathways (Figure 2A) [33,34]. The ESCRT machinery, an evolutionarily conserved family of protein complexes including ESCRT-0, ESCRT-I, ESCRT-II, and ESCRT-III, orchestrates the sorting of cargo and membrane formation to form MVBs and ILVs [35]. ESCRT-0 and ESCRT-I proteins recognize and target ubiquitinated cargo to the lipid microdomain of the MVBs membrane, while ESCRT-II and ESCRT-III facilitate the invagination and detachment processes forming MVBs and ILVs. Alternatively, the ESCRT-independent pathway utilizes lipid (ceramides)-driven [36,37] and G protein (Gi)-coupled sphingosine 1-phosphate (S1P) receptors-dependent processes to specialize cargo packaging into MVBs [38]. Newly formed MVBs may enter the lysosomal degradation pathway [39] or, dependent on the Rab GTPase family for transport and attachment to the plasma membrane, promote their fusion and subsequent exosome release through the action of soluble N-ethylmaleimide-sensitive factor attachment protein receptors (SNAREs) [40,41,42]. Subsequently, exosomes can shuttle between cells through the autocrine, paracrine, and endocrine pathways [43].

Exosome secretion is an important cellular process that occurs both in vitro and in vivo, influenced by multiple factors such as molecular motors, cytoskeletal proteins, intracellular and extracellular pH gradients, intracellular Ca^2+^ levels, and the histone deacetylase-silencing information regulator factor 1, which regulates lysosomal acidification [44,45,46,47] (Figure 2A). Additionally, organelles including the endoplasmic reticulum, Golgi apparatus, and mitochondria, or the vesicles originating from them, also play roles in exosome secretion [48,49,50]. Various stress conditions and specific organelle functions further modulate exosome formation and release. For instance, endoplasmic reticulum stress induces molecular remodeling and the subsequent formation of MVBs, leading to exosome release through the activation of stress sensors such as inositol-required enzyme 1 (IRE1) and PKR-like endoplasmic reticulum kinase (PERK) [51]. Moreover, related proteins from the Golgi apparatus such as GOLPH3 and LMAN2, along with vesicles, are associated with the content and secretion of exosomes [48,52,53]. Notably, mitochondrial-derived vesicles (MDVs) have emerged as alternative progenitors of exosomes, playing a role in mitochondrial quality control (MQC) by removing impaired mitochondrial proteins or networks [54]. Inflammatory states have been observed to significantly increase the release of MDVs [55]. A complex formed by Syntaxin-17, SNAP29, and VAMP7 orchestrated the targeting and fusion of MDVs with endosomes/lysosomes for degradation [56]. Importantly, not all MDVs are destined for lysosomes degradation; a subset distinctly merges with endosomes, contributing to MVBs formation and eventual exosomes release [57]. Proteomic studies have revealed that up to 10% of exosomes’ content is of mitochondrial origin [55,58]. Thus, organelles are important contributors to exosome secretion, providing new insights into the mechanisms that control exosome production and release.

### 2.2. Uptake and Function of Exosomes

Once released, exosomes, which include proteins, lipids, and nucleic acids, can be transported to recipient cells that recognize and capture them, thereby regulating the responses of the target cells [59]. The mechanisms of exosomes uptake vary among different receptor cells [60,61] (Figure 2B): (1) Exosomes can be endocytosed into recipient cells through various mechanisms, including clathrin-mediated endocytosis, caveolin-dependent endocytosis, lipid raft-mediated endocytosis, phagocytosis, and micropinocytosis; (2) exosomes can directly fuse with the cell membrane, delivering their contents to the cytoplasm of target cells; (3) exosomes can bind directly to cell receptors through ligands, activating downstream signaling pathways. When their contents are released inside the cell, they may regulate the function of the recipient cell either by directly changing intracellular protein levels or indirectly through nucleic acids. It is noteworthy that exosome uptake and secretion pathways may intersect, resulting in the net production of a mixed exosome population over time in any given cell, including both endogenously produced exosomes and those to be recycled. The cellular mechanisms regulating exosome uptake are not fully understood but are critically important. The different pathways associated with exosome uptake, as well as the putative specificity of exosomes for certain cell types, contribute to the complexity of exosome function in cell-to-cell communication.

**Figure 3:**
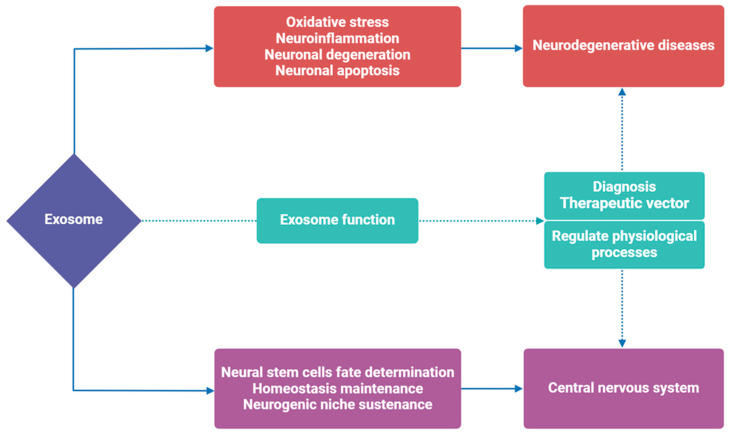
Contrasting biological functions of exosomes in the nervous system. Exosomes regulate the physiological state of the central nervous system, including the determination of neural stem cell fate, the maintenance of homeostasis, and the sustenance of neurogenic niche. Conversely, exosomes can trigger oxidative stress, neuroinflammation, neuronal degeneration, and neuronal apoptosis, further aggravating the symptoms of neurodegenerative diseases. During this process, exosomes can be used as biomarkers to diagnose disease processes or as therapeutic vectors to deliver therapeutic drugs. This figure was created with BioRender.com.

**Figure 4:**
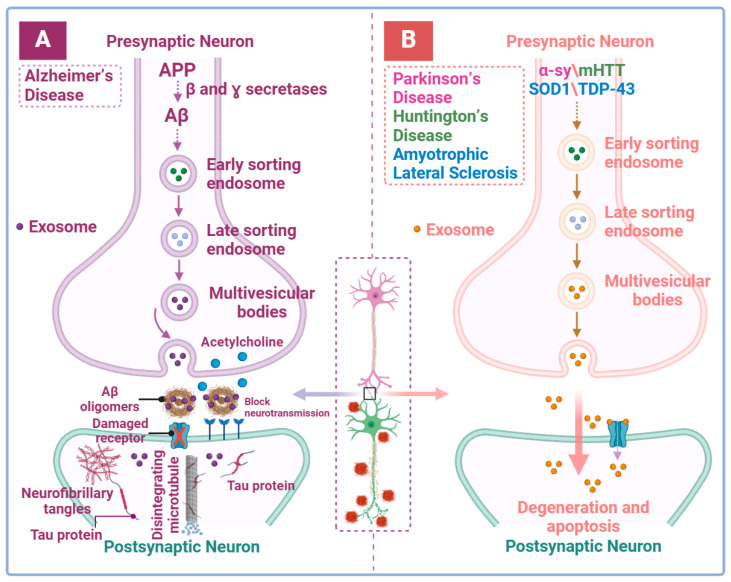
Exosomes contribute to the pathological development of neurodegenerative diseases. (**A**) Enzymatic degradation of amyloid precursor proteins to produce amyloid beta (Aβ) occurs in early-sorting endosomes (depicted by dotted lines), leading to its accumulates on exosomes. The release of exosomes facilities the formation of Aβ oligomers in the synaptic cleft, which blocks the neurotransmission of acetylcholine. Aβ is then transmitted to recipient neurons, triggering increased microtubule instability and the release of tau protein, ultimately leading to the formation of neurofibrillary tangles. These tangles induce neuronal and receptor damage, disrupting communication disruption. The combined impact of Aβ and neurofibrillary tangles results in neuronal dysfunction, contributing to the progression of AD. (**B**) Similarly, in presynaptic neurons, pathological proteins such as α-syn in PD, mHTT in HD, and SOD1 and TDP-43 in ALS are internalized into endosomes (depicted by dotted lines) and incorporated into MVBs. These exosomes are then released into the synaptic cleft, where they are taken up by recipient neurons, leading to neuronal damage and the disruption of neurotransmission. This figure was created with BioRender.com.

**Figure 5:**
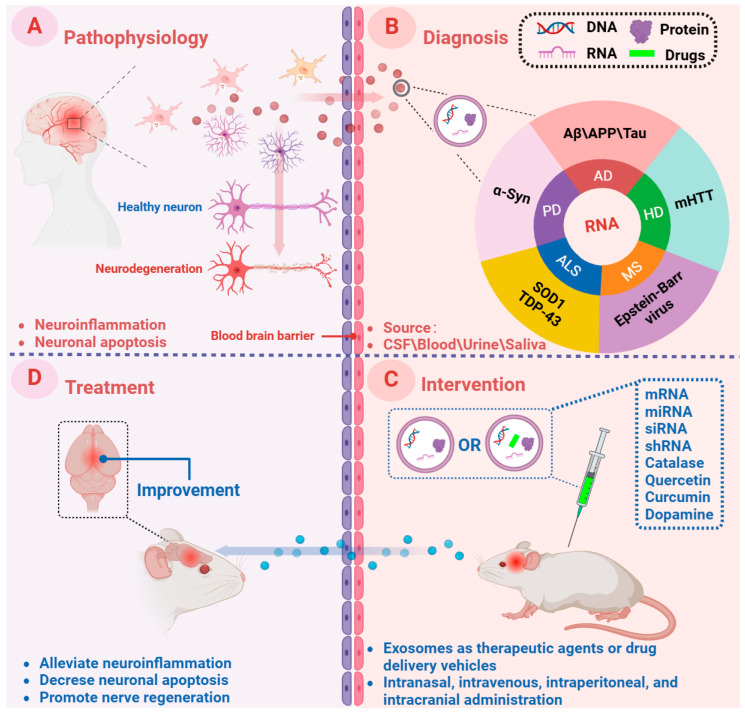
Overview of the role of exosomes in neurodegenerative diseases. (**A**) Pathophysiology: nerve cells in brain lesions release and transmit exosomes, leading to local neuroinflammation and neuronal apoptosis, etc. (**B**) Diagnosis: cerebral exosomes and exosomes that enter the peripheral circulation through the blood–brain barrier are collected as diagnostic biomarkers (derived from CSF, blood, urine, and saliva). (**C**) Intervention: in vitro, neurodegenerative diseases mouse models are treated with exosomes either directly (by themselves) or in combination with drugs (as vehicles). (**D**) Treatment: exosomes can cross the blood–brain barrier and have targeted properties to the damaged areas of the central nervous system, thus playing a therapeutic role. This figure was created with BioRender.com.

Exosomes regulate various physiological processes within the CNS, including the determination of neural stem cells fate, the maintenance of homeostasis, and the sustenance of the neurogenic niche in the adult brain [62,63] (Figure 3). Concurrently, the cargo of exosomes influences the progression of CNS diseases [6,7]. In particular, it has been elucidated that the sorting of proteins and RNA within exosomes is regulated by various pathophysiological stress stimuli and disease conditions [64,65]. For example, in NDs, increasing evidence indicates that the transmission of neurotoxic misfolded proteins via exosomes [4], such as Aβ and tau protein in AD patients, and α-syn protein in PD patients, contributes to disease propagation (Figure 4). Additionally, exosomes encapsulate small RNAs within lipid or lipoprotein complexes, protecting them from degradation during transport [66]. The transfer of circulating exosomal miRNA has been identified as a novel, highly stable mechanism of intercellular genetic communication [67]. Recent studies have shown differences in exosomal miRNAs from different cell sources and states, which play an active role in regulating neurogenesis in vitro and in vivo, thereby participating in the progression of NDs in patients and various disease models (Figure 5A,B) [67,68]. Exosomal cargo exhibits different characteristics in various NDs, suggesting that exosomes are promising potential biomarkers for the diagnosis of NDs.

Beyond their diagnostic potential, exosomes also hold significant promise as therapeutic delivery vehicles. For the therapeutic potential of drugs, proteins, or RNA to be fully realized, the development of efficient, tissue-specific, and non-immunogenic delivery technologies is imperative, and exosomes are excellent candidates for transport [17,69]. Given the unique characteristics of exosomes, their study is highly intriguing due to their capacity to deliver themselves or artificially modified therapeutic molecules to recipient cells, thereby regulating homeostasis [70,71]. Existing studies have shown that stem cell-derived exosomes themselves can intervene in the course of neurological diseases and alleviate pathological symptoms [72,73]. Exosomes can significantly enhance the efficiency of neurotherapeutic drugs entering the CNS through the peripheral circulation, thereby increasing the dose and duration of drug action [74,75]. Meanwhile, the use of exosomes for delivering the antioxidant catalase protein and mRNA across the BBB can mitigate the neurotoxicity associated with brain inflammation [18,69]. Moreover, engineered exosomes can be loaded with specific therapeutic molecules, such as siRNA [17,76] and shRNA [77], to formulate site-specific targeting strategies to cells within the brain, providing new ideas for the treatment of NDs. Therefore, the multifaceted roles of exosomes in the pathophysiological mechanism of NDs have broad application prospects, poised to significantly contribute to elucidating NDs pathogenesis, establishing efficient diagnostic methods, and developing new therapeutic techniques.

## 3. Exosomal Role in Neurodegenerative Diseases

### 3.1. Exosomes and Alzheimer’s Disease

Alzheimer’s disease (AD), the most prevalent form of dementia worldwide, is characterized by increased inflammation, oxidative stress, and neuronal loss across multiple brain regions, primarily affecting the temporal lobe and leading to hippocampus and cerebral cortex atrophy. This damage manifests as expansion of the sulci, enlargement of the ventricle, and narrowing of the gyri. The main histopathologic features of AD include the abnormal accumulation of Aβ and the presence of neurofibrillary tangles comprised of phosphorylated tau protein [78]. In AD, Aβ monomers released into the synaptic cleft form Aβ oligomers, which block neurotransmitter movement and neuronal communication [79]. Meanwhile, Aβ stimulates the phosphorylation of tau protein, detaching it from microtubules to form neurofibrillary tangles, thereby disrupting the stability of neuronal transport on the microtubule highway and leading to neuronal dysfunction [80].

Pathologically, exosomes are implicated in the release of Aβ and can exacerbate brain pathology in AD by promoting Aβ aggregation [81] (Figure 4A). It has been reported that amyloid precursor protein (APP) is localized in early-sorting endosomes, where it undergoes enzymatic processing to form Aβ. A portion of this process Aβ is embedded in the ILVs of MVBs, which are subsequently released into the extracellular space through fusion with the plasma membrane [82]. Exosomes isolated from the CSF, blood, and urine of AD mouse models have been shown to contain increased levels of amyloid precursor protein (APP, the precursor of Aβ) C-terminal fragments [83,84]. The presence of the exosomal marker protein ALIX in Aβ plaques indicates a potential role for exosomes in the formation of Aβ plaque [85]. Moreover, exosomes play a major role in the interneuronal transfer of tau and the induction of AD. Exosomes can deliver phosphorylated tau protein, thereby promoting neurotoxicity [86]. Inhibition of exosome synthesis has also been shown to significantly reduce tau proliferation [87]. Existing studies have also shown that in AD, imbalance of the extracellular exosomal RNA content may promote the disease progression and, in conjunction with Aβ and tau proteins, contributes to the clinical recognition and evaluation of AD (Table 1).

Relative to pathological diagnosis, reducing the neurotoxicity of AD through exosomes is a more novel approach (Figure 5C,D). Sustained injection of neuroblastoma and neuron-derived exosomes into the brains of APP transgenic mice have demonstrated the ability to capture Aβ, significantly lower Aβ levels and thus reducing the pathological burden of AD [85,122]. Similar reports have shown that exosomes from hypoxia-preconditioned mesenchymal stromal cells ameliorate cognitive decline in APP transgenic mice by reducing Aβ accumulation and amyloid plaque formation, rescuing synaptic dysfunction, and regulating inflammatory responses [123]. Additionally, exosomes isolated from mesenchymal stem cells (MSCs) or adipose-derived stem cells have demonstrated disease-regulating effects by clearing Aβ deposits in nerve cells or the brain, reducing neuroinflammation, and preventing neuronal death [124,125]. As mentioned above, exosomes with pathological states are involved in the pathogenesis of AD, while exosomes of normal physiological origin show protective effects against AD progression. Existing studies have also indicated that exosomes can be manipulated to manage AD as needed.

Exosomes target the foreign portion of the peripheral circulation to the brain through fusion, receptor-mediated transport, and intracellular and transcellular mechanisms [126], thereby improving drug delivery efficiency and therapeutic effectiveness. For example, quercetin, known for its poor brain targeting and low bioavailability, has limited efficacy in diseased brain regions [74]. Intravenous injection of plasma-derived exosomes can inhibit cyclin-dependent kinase 5 (CDK5)-mediated tau phosphorylation and reduce the formation of insoluble neurofibrillary tangles in AD mice by improving the bioavailability and brain delivery efficiency of quercetin [74]. Similarly, oral administration of exosomal curcumin to female Sprague-Dawley rats resulted in several-fold higher levels of the drug in the brain compared to dietary intake of curcumin [75]. Exosomes isolated from curcumin-treated macrophages (RAW264.7) exhibit specific active targeting between lymphocyte function-associated antigen 1 (LFA-1) and endothelial intercellular adhesion molecule 1 (ICAM-1), increasing drug permeability to the BBB. Intraperitoneal injection of these exosomes activates the AKT/GSK-3β pathway in the brain to inhibit tau phosphorylation, effectively preventing neuronal death and alleviating AD symptoms both in vitro and in vivo [127]. In addition, given that exosomes naturally carry RNA, it is hypothesized that they can deliver small RNAs to the brain to treat AD. Dendritic cell-derived exosomes, functionalized with neuron-specific rabies viral glycoprotein (RVG) peptide expressing Lamp2b, have been used to specifically deliver siRNA against BACE1, a therapeutic target for AD, to neurons, microglia, and oligodendrocytes in the brain, resulting in the specific knockdown of BACE1 [17]. Thus, exosomes hold great potential in reducing AD toxic protein deposition, improving drug and gene targeted delivery and therapeutic efficiency, and restoring neuronal function.

### 3.2. Exosomes and Parkinson’s Disease

Parkinson’s disease (PD) is the second most common ND after AD [128]. The key molecular and neurophysiological mechanisms of PD pathology involve the intra-neuronal aggregation of misfolded α-syn and the presence of Lewy bodies [16]. Lewy bodies, characterized by the aggregation of oligomeric α-syn, represent the main neuropathological change in PD [16]. The mechanisms through which aggregated α-syn exerts neurotoxicity include mitochondrial defects, proteasome impairments, endoplasmic reticulum stress, and inflammatory responses [129].

Exosomes facilitate the dissemination of a-syn and inoculate early molecular changes in PD pathology into nerve cells. MVBs containing α-syn, formed in association with endosomes, contribute to the transmission of PD pathology following fusion with the plasma membrane (Figure 4B) [130,131]. In human neuroblastoma SH-SY5Y cells, the release of exosome-associated α-syn occurs in a calcium-dependent manner (regulator of exosome secretion), resulting in cell death and indicating that α-syn aggregation is closely related to exosome secretion [132]. Exosomes enable both monomeric and oligomeric α-syn to mediate prion-like [133], longer-distance, and more efficient propagation between various nerve cells such as microglia [134], astrocytes [135], and neurons [135], leading to neurotoxicity. In addition, α-syn degradation proceeds through the ubiquitin-proteasome system (UPS) and the autophagy-lysosomal pathway (ALP) [136]. Inhibition of the ALP pathway promotes a compensatory enhancement of the MVBs and amphisome pathways, thus establishing the cytoprotective effect of exosomes by reducing α-syn levels within neurons through the release of exosomes [137,138,139]. However, while the compensatory mechanism for α-syn clearance in cells is strengthened by exosome release to avoid its intracellular accumulation, this procedure exacerbates PD pathology and may lead to an overload of toxic α-syn in the extracellular space [140]. Additionally, miRNAs packaged in exosomes have been shown to be associated with PD, regulating key pathogenic pathways including autophagy, inflammation, and protein aggregation [141]. Therefore, the classification of exosome cargo not only provides a theoretical basis for understanding the etiology of PD but also plays a role in the pathogenesis of PD and is applicable to pathological analysis (Table 1).

In addition, researchers have explored using exosomes as specific transporters to deliver bioactive substances in response to PD (Figure 5C,D). The exosome delivery system crosses the BBB through the interaction between transferrin and the transferrin receptor, delivering fully saturated dopamine (DA) to the brain [142]. This results in a more than 15-fold increase in DA distribution within the PD brain [143], indicating exosomes have natural targeted delivery capabilities. In a mouse model of PD, DA-loaded exosomes showed better therapeutic efficacy and lower systemic toxicity compared to the intravenous administration of free dopamine [143]. Additionally, PD in vitro and in vivo trials have shown that exosome delivery and uptake of the antioxidant catalase protein or mRNA increased neuronal survival and reduced neurotoxicity and neuroinflammation [18,69]. In the 6-OHDA mouse model of PD, MSCs-derived exosomes proved effective in rescuing dopaminergic neurons. They can also carried miRNAs and interacted with neuronal cells, thereby reducing neuroinflammation and promoting neurogenesis [72]. In genetic modification, siRNA is considered a promising therapeutic approach. In transgenic mice expressing the human phosphorylated mimic S129D α-syn, systemic injection of modified exosomes expressing RVG peptide loaded with siRNA significantly reduced α-syn mRNA and protein levels in the brain [76]. Therefore, exosomes can overcome natural barriers and effectively improve the stability and targeting of siRNA. However, due to the short efficacy of siRNA and its low bioavailability in the systemic circulation, its application in neurological diseases remains limited. Addressing this, designing RVG-exosomes to bind shRNA minicircles targeting α-syn in the brain of PD mice can reduce α-syn aggregation and loss of dopaminergic neurons, improving the pathological symptoms of PD [77]. These studies support the use of exosomes as transport vehicles for active molecules (such as enzyme) or genetic regulators (including mRNA, miRNA, siRNA, and shRNA), by enhancing their stability in circulation and greatly increasing their therapeutic potential in clinical applications.

### 3.3. Exosomes and Huntington’s Disease

Huntington’s disease (HD) is a progressive, dominant hereditary ND caused by an abnormal expansion of CAG repeats in the huntingtin gene (HTT). The number of CAG repeats is inversely correlated with the age of onset; the greater the number of repeats is, the earlier the onset of the disease is, and vice versa [144]. Genetic diagnosis can be used to detect the number of CAG repeats in affected and high-risk individuals [145]. This genetic mutation leads to the production of mutant Huntington (mHTT) protein, which accumulates within brain cells and exhibits significant neurotoxicity. Typical symptoms of HD include cognitive impairment, neuropsychiatric issues, and involuntary choreiform movements [146]. Although HD is currently incurable, identifying early diagnostic clues or biomarkers that reflect disease progression remains crucial for patient care.

The transfer of mHTT between neurons may occur via tunneling nanotube and/or vesicular mechanisms [114], suggesting that exosomes are likely involved in its transmission (Figure 4B). However, while changes in exosomal proteins have been extensively studied in more common NDs such as AD and PD, less is known about their role in HD [147] (Table 1). Exosomes may carry mHTT and its fragments or other proteins reflecting the cellular state at the time of exosomes production [114], making them potential biomarkers for HD. In addition, disease-associated amplified RNAs found in the body fluids of HD patients appear to be prime candidates for monitoring disease progression [148]. Previous studies have analyzed differences in miRNA expression in CSF between healthy individuals and presymptomatic HD gene expansion carriers, as well as in plasma from HD patients, showing promise for early detection and diagnosis of HD [149,150]. However, these studies did not address differences in the content and composition of exosomal miRNAs, suggesting that further research is needed to elucidate their potential role. On the other hand, exosomes may also provide therapeutic support for HD (Figure 5C,D). Exosomes secreted by astrocytes and adipose-derived stem cells have demonstrated neuroprotective effects. Injection of astrocyte-derived exosomes into the striatum of HD 140Q knock-in (KI) mice has been shown to reduce the density of mHTT aggregates and potentially alleviate HD pathology [151]. Similarly, exosomes from adipose-derived stem cells, which release neurotrophic factors, have been shown to reduce mHTT aggregation, mitochondrial dysfunction, and apoptosis in an in vitro HD model [152].

In HD, exosomes are particularly effective at delivering oligonucleotides such as miRNA and siRNA. For instance, exosomes derived from HEK293 cells overexpressing miR-124 were injected into the striatum of R6/2 transgenic HD mice. These exosomes contained miR-124, which reduced the expression of the target gene RE1-Silencing Transcription Factor [153] and promoted neuronal differentiation and survival [154]. However, this intervention did not result in significant behavioral improvements, indicating the low therapeutic efficacy of exosomal miR-124. Enhancing the therapeutic impact may require selectively increasing the dose or altering the type of miRNAs in the exosomes. Additionally, exosomes expressing RVG peptide combined with siRNA targeting the human huntingtin exon 1 (HuHtt) transcript significantly reduced HuHtt mRNA and protein levels in the brains of BACHD and N171-82Q mouse models [155]. To improve delivery efficiency, researchers synthesized hydrophobically modified siRNA (hsiRNA) by binding cholesterol to siRNA, enhancing stability, and promoting cellular internalization [156,157]. This method enabled exosome-delivered cholesterol siRNA to induce HTT mRNA silencing in neurons more effectively than cholesterol siRNA alone. In another study, hsiRNA targeting HTT mRNA was efficiently loaded into exosomes upon co-incubation without altering vesicle size distribution or integrity. In vitro, these exosomes mediated the internalization of hsiRNA–HTT into primary cortical neurons, resulting in dose-dependent silencing of HTT mRNA and protein. Unilateral infusion of these exosomes into the mouse striatum resulted in significant bilateral silencing of up to 35% of HTT mRNA [158,159]. Therefore, exploiting exosome affinity for specific cell may extend the way for the effective delivery of specifically targeted oligonucleotides in the treatment of HD. Nonetheless, despite the potential reference value of exosomes for the diagnosis and treatment of HD, there are limitations due to the inestimable exosome loads and the relative scarcity of delivery methods. The possibility of using exosomes as novel therapeutic vectors for HD, especially the as biomarkers, is still in its early stage, and more research is needed in this field.

### 3.4. Exosomes and Amyotrophic Lateral Sclerosis

Amyotrophic lateral sclerosis (ALS) is a severe neurological disease characterized by symptoms that include muscle weakness, paralysis, atrophy, and eventually respiratory failure leading to death. Beyond motor system dysfunction, approximately 50% of ALS patients may also exhibit psychiatric disorders [160]. The pathophysiological mechanism of ALS involves complex pathways, including mitochondrial dysfunction, RNA processing, glutamate excitotoxicity, endoplasmic reticulum stress, protein homeostasis, and endosomal transport, as well as EV secretion [161,162,163]. ALS is marked by the dysfunction of proteins, notably those devoid of nucleic acid components [160,164], with the cortex, brainstem, and spinal cord motor neurons primarily affected by the prion-like misfolded proteins SOD1 and TDP-43, which lead to neurodegeneration.

Exosomes may facilitate the accumulation of SOD1 and TDP-43 in cells, implicating exosomes as an important component in the pathological development of ALS [165] (Figure 4B). Research has shown that NSC-34 motor neuron-like cells, whether overexpressing wild-type or mutant SOD1, can secrete SOD1 via exosomes [166]. Additionally, in NSC-34 cells, both misfolded mutants and wild-type SOD1 proteins have been observed to transfer between cells via exosomes and protein aggregates [167]. Research has demonstrated that astrocyte derived exosomes can efficiently transport misfolded SOD1 proteins to adjacent spinal cord neurons, inducing motor neuron death [168]. Furthermore, exosomes derived from ALS brain tissue have been shown to promote TDP-43 aggregation in human neuroblastoma SH-SY5Y cells, indicating a key role of exosomes in transporting TDP-43 aggregates [169]. Exposure of Neuro2a cells to exosomes from ALS brain resulted in cytoplasmic redistribution of TDP-43 [170]. Interestingly, inhibition of exosome secretion by inactivation of neutral sphingomyelinase 2 with GW4869 or by silencing RAB27A led to the formation of TDP-43 aggregates in Neuro2a cells. In addition, administration of GW4869 exacerbated the disease phenotypes in transgenic mice expressing the TDP-43A315T mutant [170]. These findings suggest that exosomes not only facilitate the proliferation of TDP-43 aggregates but also potentially aid in their constructive elimination, complicating therapeutic strategies that solely focus on exosome inhibition. Notably, in ALS, TDP-43-carrying exosomes not only shuttle between nerve cells but also interact with monocytes in the peripheral circulation and participate in immune regulation, further supporting the hypothesis that prion-like transmission promotes spatiotemporal progression of ALS [171]. Meanwhile, other exosomal components, including miRNAs delivered to the extracellular space or peripheral circulation, are also very effective in promoting ALS progression or detecting disease development (Table 1).

In recent years, the application of stem cell-derived exosomes in the treatment of ALS has emerged as a promising area of research (Figure 5C,D). Researchers have treated an in vitro cellular model of ALS using adipose-derived stem cell (ASC)-derived exosomes (ASC-Exo). This treatment was found to repair mitochondrial dysfunction and restore mitochondrial membrane potential, suggesting that ASC-Exo could be effective in treating diseases characterized by mitochondrial adaptation [73]. Subsequently, experiments were conducted where ASC-Exo was injected into SOD1 transgenic (G93A) ALS mice. This intervention significantly improved motor performance and conferred protection to lumbar motor neurons, neuromuscular junctions, and muscles [172]. Additionally, exosomes isolated from bone marrow MSCs have also been shown to reduce damage in brain cell models in ALS mice [173]. By treating bone marrow MSCs with interferon-γ, the upregulation of miR-466q and miR-467f in exosomes inhibit the p38 mitogen-activated protein kinase pathway, decrease the expression levels of tumor necrosis factor and interleukin 1b, and alleviate the inflammatory activation of SOD1-G93A microglial cells. Currently, research focusing on the isolation and therapeutic application of exosomes from totipotent stem cells is expanding, demonstrating preliminary value in addressing ALS.

### 3.5. Exosomes and Multiple Sclerosis

Multiple sclerosis (MS) is an autoimmune ND that targets the CNS. MS is caused by the migration of autoreactive lymphocytes to the CNS, triggering an inflammatory response that leads to varying degrees of demyelination, axon loss, and gliosis [174,175,176]. Common symptoms of MS include sensory disturbances (such as loss of sensation, paresthesia, dysesthesias, and tingling), visual disturbances (such as double vision, vision loss), motor disturbances (including focal and limb weakness, fatigue, tremor), bladder and bowel incontinence, and cognitive dysfunction [174,175].

Recent studies have highlighted the key role exosomes play in MS. Studies have shown that in MS patients, exosomes not only transport specific markers (Table 1) associated with the disease [5,177] but are also involved in activating immune cells and influencing the inflammatory response within the CNS. From the perspective of MS mechanism, exosomes play an important role in immune cell migration across the BBB. In addition to white blood cells, microglia, astrocytes, and platelets, endothelial cells are also found to secrete exosomes containing metalloproteinases and caspase-1 under the stimulation of pro-inflammatory cytokines such as interferon-γ (IFN-γ), tumor necrosis factor (TNF), and interleukin-1 (IL-1). These exosomes induce BBB disruption, promoting the migration of lymphocytes and myeloid cells into the CNS [178,179]. Furthermore, exosomes shed from platelets of MS patients have been found to express P-selectin, a receptor for PSGL-1 and PECAM-1 on lymphocytes. P-selectin signaling leads to elevated expression of the integrin alpha4-beta1 on lymphocytes, which facilitates their adhesion to endothelium and subsequent transmigration [180,181]. Another mechanism known to enhance the immune cells migration to the CNS relies on the transfer of integrin Mac-1 (a receptor for ICAM-1) from endothelial exosomes to monocytes, thereby increasing their ability to cross the endothelial barrier [181]. After gaining entry into the CNS, the activated immune cells can release a cascade of inflammatory cytokines into the extracellular. Subsequently, activated CNS cells, in turn, release EVs, further enhancing neuroinflammation and neurodegeneration in MS. For example, reactive astrocytes can secrete microvesicles containing matrix metalloproteinases (MMPs) [182], further disrupting the BBB and exacerbating neuroinflammation. Activated macrophages transported to the brain as well as resident microglia can release EVs containing IL-1β [183], major histocompatibility complex class II (MHC II) molecules [184], and other cargo, further spreading inflammatory signals [185]. Taken together, these data suggest that exosomes facilitate immune cell transport across the BBB, thereby contributing to this critical pathogenic step in the development of MS.

Currently, the treatment of MS patients mainly focuses on immune system regulation and suppression strategies. In addition to their role in immune response suppression, some of these treatments have shown potential to promote partial or complete myelin regeneration, emphasizing the need for new therapeutic pathways (Figure 5C,D) [186]. One promising pathway involves MSCs-derived EVs, particularly exosomes. These EVs are considered to be potentially safer alternatives to MSCs in therapeutic approaches, offering the benefits of MSCs therapy while minimizing some of the associated risks [187,188]. MSCs-derived EVs have been shown to alter the polarization of macrophages and microglia from the pro-inflammatory M1 phenotype (producing TNF-α, IFN-γ, and IL-12) to the anti-inflammatory M2 phenotype (upregulating IL-10). This neuroprotective phenotype may alleviate disease progression by reducing inflammation and demyelination of the brain and spinal cord [189,190,191]. In addition, studies have shown that MSCs-derived EVs downregulate the production of IL-22 and IL-23, induce an anti-inflammatory phenotype of macrophages called regulatory macrophages, and that immunomodulatory changes in the brain microenvironment inhibit the pathogenic T helper 17 (Th17) responses [192]. In addition, the study found that when aged animals were exposed parabiotically to a youthful systemic milieu, the differentiation of their oligodendrocyte precursor cells could be stimulated, thereby improving remyelination [193]. Moreover, miR-219 was enriched in serum-derived exosomes in young animals, and intranasal administration of these exosomes in aged rats significantly increased the levels of myelin, neural stem cells, and oligodendrocyte precursor cells, while reducing oxidative stress [194]. Exosomes from several circulating immune cells can also increase the myelin content and reduce oxidative stress in slice cultures, and these exosomes all contain miR-219 [195]. Meanwhile, an in vitro study revealed that IFNγ-DC-exosomes containing miR-9 and miR-17-92 cluster, which are involved in the oligodendrocyte differentiation pathway, increased myelination and oxidative resistance [196]. These studies highlight the potential therapeutic role of exosomal miRNAs in treating patients with MS.

## 4. Conclusions and Prospects

Exosomes are ubiquitous in all bodily fluids and possess the capability to encapsulate complex molecules from both inside and outside the cell. Consequently, they emerge as formidable candidates for minimally invasive liquid biopsies, offering the prospect of monitoring disease progression through longitudinal sampling. In addition, the presence of specific proteins on the surface of exosomes enhances their immunocapture and enrichment, making them invaluable for integrated multiparameter diagnostic assays. Their intricate biogenesis pathways and specialized loading mechanisms, adapted to different physiological and pathological conditions, designate these vesicles as essential mediators of a broad spectrum of signals, ranging from protective to pathological.

The ongoing investigation into exosomes, both as independent entities and as vectors for drug delivery, highlights their therapeutic potential. However, the reliability of exosome-based diagnosis and treatment depends on numerous factors, including sample collection, storage conditions, exosome isolation techniques, precipitation methods, biomarker identification procedures, drug loading patterns, and delivery routes. Despite advancements facilitating exosome research for clinical diagnosis and model therapy, challenges such as low yield, poor drug load rates, lack of reproducibility, sample contamination, and labor-intensive processes persist in this domain. Moreover, the diversity of biological samples employed and the lack of standardized detection and analysis methods contribute to inconsistent results across studies, detracting from the diagnostic, therapeutic, and mechanistic significance of these discoveries. Therefore, the establishment of universally accepted standard methodologies is imperative to validate diagnostic methods and facilitate their clinical application.

The current understanding and research on exosomes primarily concentrate on their role in regulating intercellular communication through cell signaling and content transport, thereby promoting the onset and progression of diseases. Extensive research on exosomes in NDs has been directed toward understanding pathophysiological mechanisms, disease progression, biomarkers, and therapeutic approaches. However, aside from clinical studies of biomarkers derived from CSF, blood, saliva, and urine, the bulk of neurological research relies on animal models or in vitro cell culture data. Particularly, the safety and technological challenges of clinical treatment with exosomes remain significant hurdles. It is important to recognize that not all components of therapeutic exosomes have biological functions to ameliorate NDs pathologies or promote beneficial physiological transformations. Understanding the cargo sorting mechanism of exosomes is crucial for leveraging their potential as therapeutic carriers. Moreover, advancing exosomes as a drug delivery model needs to expand their practicability and applicability. A paramount challenge in exosome therapy is effectively utilizing their stability and high affinity to traverse the BBB, maximizing safe delivery efficiency and superior targeting capabilities to intervene in disease processes while mitigating associated risks. Therefore, to reveal the multifaceted biological role of exosomes in NDs, a comprehensive and meticulous method to their identification and functional evaluation is imperative.

Moving forward, advanced methods for isolating and characterizing exosome with high purity will be critical. The establishment of standardized protocols, or “gold standards”, are urgently needed to accurately distinguish exosomes from contaminating proteins, lipoprotein aggregates, and other extracellular vesicles. When applying exosome research to human disease, large-scale, muti-cohort studies across diverse patient populations will also be essential. Such population-level investigations are needed to fully realize the potential of exosomes as clinical diagnostic biomarkers and therapeutic vectors for NDs. These studies will ensure the reproducibility and generalizability of findings, enhancing both the sensitivity and specificity of exosome classification. This will enable more accurate pathological analysis and personalized interventions tailored to individual patients’ specific clinical conditions. Ultimately, precision medicine approaches leveraging exosome cargo characterization have the potential to lead to more targeted disease management strategies and improved therapeutic outcomes for neurodegenerative patients.

## Figures and Tables

**Table 1:** Summary of exosomal components in body fluids across neurodegenerative diseases.

Neurodegenerative Diseases	Exosomes Source	Exosome Cargo	References
Alzheimer’s disease	CSF	APP C-terminal fragments, p-tau, p-T181-tau, miR-193b, miR-451a, miR-135a, miR-16-5p	[83,84,88,89,90,91,92]
	Plasma	A0A0G2JRQ6, C1QC, CO9, GP1BB, RSU1, ADA10, LncRNA BACE-AS1, LncRNA NEAT1, LncRNA GAS5, LncRNA MALAT1, Aβ42, t-tau, p-tau181	[93,94,95]
	Serum	miR-384, miR-193b, miR-135a	[91,96]
	Blood	APP C-terminal fragments, Aβ1-42, tau, p-T181-tau, p-S396-tau, miR-193b, miR-451a, neurogranin	[83,84,89,90,97,98,99]
	Urine	APP C-terminal fragments	[83,84]
Parkinson’s disease	CSF	miR-331-5p, miR-505	[100,101,102]
	Plasma	α-syn, apolipoprotein A1	[100,103,104,105,106,107]
	Serum	miR-19b, miR-24, miR-195	[108]
	Blood	α-syn	[100,109,110,111]
	Saliva	α-syn	[112]
	Urine	miR-184	[113]
Huntington’s disease	Plasma	mHTT	[114]
Amyotrophic lateral sclerosis	CSF	miR-124-3p	[115]
	Plasma	miR-15a-5p, miR-193a-5p	[116]
	Serum	miR-27a-3p	[117]
	CSF	miR-132-5p, miR-320a-5p	[118]
Multiple sclerosis	Serum	Epstein-Barr virus protein, hsa-miR-122-5p, hsa-miR-196b-5p, hsa-miR-301a-3p, hsa-miR-532-5p, miR- 15b-5p, miR-451a, miR-30b-5p, miR-342-3p, miR-127-3p, miR-370-3p, miR-409-3p, miR-432-5p, acid sphingomyelinase, myelin oligodendrocyte glycoprotein	[5,119,120,121]

## Abbreviations

CNS, central nervous system; NDs, neurodegenerative diseases; AD, Alzheimer’s disease; PD, Parkinson’s disease; HD, Huntington’s disease; ALS, amyotrophic lateral sclerosis; Aβ, amyloid-beta; α-syn, alpha-synuclein; HTT, mutant huntingtin; CSF, cerebrospinal fluid; BBB, blood–brain barrier; EVs, extracellular vesicles; MVBs, multivesicular bodies; MVs, microvesicles; ESEs, early-sorting endosomes; LSEs, late-sorting endosomes; ILVs, intraluminal vesicles; TSG101, tumor susceptibility gene 101; ALIX, apoptosis-linked gene 2-interacting protein X; IRE1, inositol required enzyme 1; PERK, PKR-like endoplasmic reticulum kinase; MDVs, mitochondrial-derived vesicles; MQC, mitochondrial quality control; APP, amyloid precursor protein; CDK5, cyclin-dependent kinase 5; LFA-1, lymphocyte function-associated antigen 1; ICAM-1, endothelial intercellular adhesion molecule 1; RVG, rabies viral glycoprotein; UPS, ubiquitin-proteasome system; ALP, autophagy-lysosomal pathway; DA, dopamine; HTT, huntingtin gene; mHTT, mutant Huntington; HuHtt, human huntingtin exon 1; hsiRNA, hydrophobically modified siRNA; ALS, amyotrophic lateral sclerosis; ASC, adipose-derived stem cell.
